# Internet-Based Cognitive Behavioural Therapy for Individuals With Depression and Chronic Health Conditions: A Systematic Review

**DOI:** 10.7759/cureus.37822

**Published:** 2023-04-19

**Authors:** Dipannita Adhikary, Shanto Barman, Redoy Ranjan

**Affiliations:** 1 Department of Family Medicine, The University of Edinburgh, Edinburgh, GBR; 2 School of Medicine, Mugda Medical College and Hospital, Dhaka, BGD; 3 Department of Surgical Science, The University of Edinburgh, Edinburgh, GBR; 4 Department of Biological Science, Royal Holloway, University of London, London, GBR; 5 Department of Cardiac Surgery, Bangabandhu Sheikh Mujib Medical University, Dhaka, BGD

**Keywords:** cognitive behavioural therapy (cbt), psychodrama and group therapy, cognitive behavioural therapy, intervention and psychotherapy for children and adolescents, internet-based cognitiive behavioural therapy, cognitive-behavioral therapy, cognitive behavioral therapy-diabetes, cognitive and behavioral therapy, efficacy- cognitive-behavioral therapy, efficacy- cognitive-behavioral therapy -major depressive disorder

## Abstract

Cognitive behavioural therapy (CBT) has heightened the need for internet-delivered intervention for depression with chronic diseases than a traditionally-based treatment procedure, and the need for CBT as an internet-delivered intervention has increased because it scales down the stigma of proceeding to a therapist, saves travel time from different geographical areas, and increases access to the service. This study aimed to evaluate the contemporary evidence for the effectiveness of internet-delivered cognitive behavioural therapy (CBT) as a treatment option for depression with chronic illness (CVD, diabetes, chronic pain, cancer, and chronic obstructive pulmonary disease (COPD)) among adult populations in high-income countries. A systematic search strategy was devised based on selecting search terms, inclusion and exclusion criteria, and refinement processes. Electronic searches were conducted using databases related to healthcare and containing peer-reviewed literature: the Cumulated Index to Nursing and Allied Health Literature (CINAHL), the Excerpta Medica Database (Embase), the Medical Literature Analysis and Retrieval System Online (Medline), and PsycINFO. Key search terms were applied to all databases and combined using Boolean operators to maximise the efficiency of the search. This review included randomised controlled trials (RCTs) evaluating the adult population aged ≥18 years published from 2006-2021. The Preferred Reporting Items for Systematic Reviews and Meta-Analyses (PRISMA) statement was employed to guide the review process. The initial search yielded 134 studies across all databases, which were refined, leading to 18 studies in the final review data set. This review suggests that internet-based CBT is an effective strategy for reducing depressive symptoms in patients with comorbid depression and chronic diseases.

## Introduction and background

Chronic disease accounts for approximately 30% of all deaths worldwide [1, 2]. Depression and anxiety disorders are the leading cause of disability around the world and contribute to the chronic global disease burden [3-6]. Furthermore, depression may be caused by a complex relationship between biological, psychological, and social factors such as unemployment, grief, bereavement, and childhood experiences [5-8]. Evidence suggests that approximately 60% of physical illness has a confounding psychological contribution, although it is often difficult to detect and diagnose [4-6]. While there is pharmacological and psychological management of different treatments for depression of proven value, in low- and middle-income countries (LMICs), the supportive services patients need are often underdeveloped or absent, despite a very high prevalence of these conditions [7-9]. Moreover, the relationship between chronic diseases and psychological distress has been neglected globally. Early psychiatric research found its potential to increase the length of hospital stays, increase hospital visits, decrease health service and treatment implementation, and delay returning to the workplace [9,10].

Many published studies have shown the beneficial effects of cognitive behavioural therapy (CBT) on mental health problems, including anxiety and depression [10,11]. Internet-delivered cognitive behavioural therapy (ICBT) has become one of the most effective forms of psychological treatment in the modern world. Recent developments like audio-visual forms and availability in CBT have heightened the need for internet-delivered intervention rather than traditional treatment delivery approaches; amongst other benefits, they may save patients and therapists significant waiting time [12]. The audio-visual form is preferred to attract clients irrespective of age, gender, and language. Moreover, it is accessible to a large proportion of the population compared with traditional forms of treatment [13-15]. ICBT also reduces the stigma of seeing a therapist and reduces travel time from different geographical areas; indeed, internet-delivered CBT can motivate patients at a reduced cost [16-17]. To date, ICBT has been mainly available in high-income countries, and several trials have examined the impacts of CBT interventions through randomised trial research. Although extensive research has been conducted, it is still being determined whether internet-based CBT is equally convincing when other health problems (beyond mental health) are addressed. Furthermore, it may not be appropriate for illiterate populations and can lead to misunderstandings between clients and practitioners [18-20].

This study aimed to evaluate the contemporary evidence and conducted an updated systematic review focused on the effectiveness of ICBT as a treatment option for depression with chronic illness among adult populations in high-income countries.

## Review

Materials and methods

Data Sources and Search Strategy

**Figure 1:**
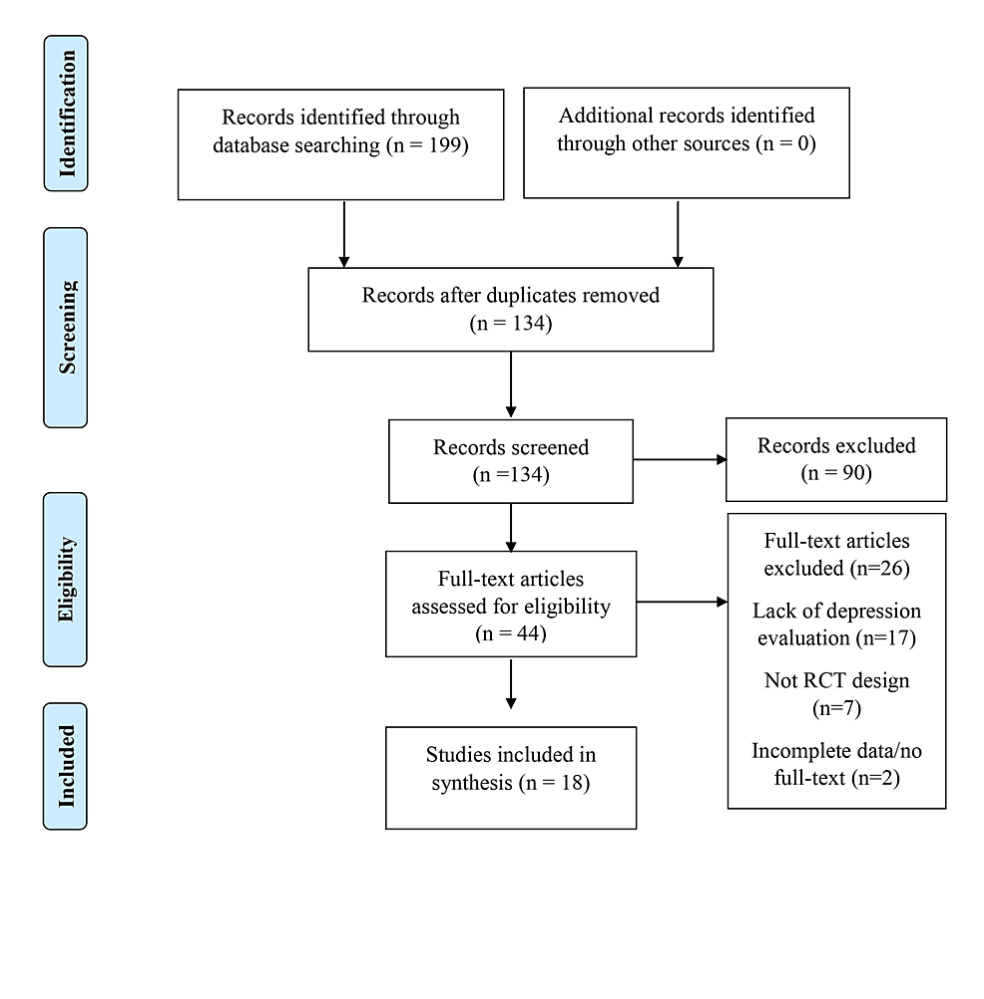
A PRISMA flow diagram that showcases the search strategy and the review process

A systematic search strategy was devised based on selecting search terms, inclusion and exclusion criteria, and refinement processes [17]. The Preferred Reporting Items for Systematic Reviews and Meta-Analyses (PRISMA) statement was employed to guide the review process (Figure 1) [20]. Electronic searches were conducted using databases related to healthcare and containing peer-reviewed literature: the Cumulated Index to Nursing and Allied Health Literature (CINAHL), the Excerpta Medica Database (Embase), the Medical Literature Analysis and Retrieval System Online (Medline), and PsycINFO. Key search terms were applied to all databases and combined using Boolean operators to maximize the efficiency of the search. Search terms were derived from the keywords of articles used in the background literature, reflecting reviewer-generated synonyms and additional terms derived from Medical Subject Headings (MeSH) criteria. The search term was "chronic disease", OR "diabetes", OR "COPD", OR "chronic pain", OR "cancer", OR "cardiovascular disease", OR "Internet CBT", OR "iCBT", OR "online CBT", OR "depression", OR "anxiety". Furthermore, we have utilized filters to search publications between 2006 and 2021 with only "English language" AND "primary research" AND "RCT".

Inclusion and Exclusion Criteria

Inclusion and exclusion criteria were selected to ensure the relevance and quality of identified studies [17]. This review included only randomised controlled trials (RCTs), as these studies represent the most reliable and unbiased method of appraising the efficacy of a clinical intervention according to the evidence hierarchy [18]. Other forms of research were excluded, including observational studies, non-randomised trials, quasi-experimental studies, case series, case studies, or review studies. Studies were only included if published from 2006-2021 to reflect contemporary literature. Albeit, studies could be based on participants from any nation; however, we preferred only high-income countries according to the World Bank country classifications to maximise relevance to healthcare settings in the United Kingdom. However, only studies published in English were included to avoid translation costs and reduce the risk of introducing translation errors into the review findings.

A focus on human adults (18 years of age or older) was preferred to minimise the bias potentially caused by paediatric populations in the data set; they may have different treatment experiences and outcomes linked to CBT use [19]. The review focused on digital interventions for patients with concurrent depression and chronic illnesses; specific illnesses associated with a risk of depression were explicitly included, reflecting the strategies employed in previous reviews [10]. All patients with chronic diseases had received a formal diagnosis or met appropriate diagnostic criteria within the included studies to be eligible for the review.

Quality Assessment

The Critical Appraisal Skills Programme (CASP) tool for RCTs was employed to guide objective, formal critical appraisal of the identified literature [21]. This tool is recognised as a standard and reliable method for appraising essential features related to bias in the design of RCTs and is amenable to use by a novice reviewer. The findings of the CASP appraisal were used to inform a critical appraisal of the literature following data synthesis.

Data Extraction and Synthesis

Data extraction was completed using a standard tabulated approach, whereby critical data from each study was identified and noted for simple comparison. Critical data included study authors and dates, study intervention characteristics, the population studied, outcomes assessed, and crucial findings. All data were extracted by a single author using this standardised approach. A narrative synthesis was performed following data extraction, as heterogeneity in study designs was considered obstructive to the completion of analytical methods such as meta-analysis [22].

Results 

Search Findings and Overview of the Included Studies

The initial search process yielded 134 studies across all databases, which were refined, leading to 18 studies in the last review data set [4,12,13,23-37]. The characteristics of the included studies are presented in Table 1. The primary reasons for excluding studies included a lack of concurrent depression and chronic disease in the population sampled, failure to adopt a straightforward RCT methodological design, incomplete data reporting, or the availability of abstracts only. The included studies evaluated the use of ICBT in the context of chronic pain (n=5), cardiovascular disease (n=5), cancer and cancer survivorship (n=6) and diabetes (n=2); no studies that focus on ICBT use in concurrent COPD and depression have been identified. All studies adopted an RCT design, although there was variability in the population sizes, sampling methods, and outcome measures, as noted in Table 1. The existence of additional sources of heterogeneity between studies is considered within the narrative analysis according to the specific chronic disease analysed.

**Table 1:** Summary of the study characteristics ACT: acceptance and commitment therapy; CVD: cardiovascular disease; ICBT: internet-based cognitive behavioural therapy; MI: myocardial infarction; ODF: online discussion forum; QoL: quality of life; TAU: treatment as usual

Author and date	Chronic disease	CBT intervention	Comparator	Sample	Outcomes	Findings
Glozier et al., 2013 [4]	CVD (high risk); Country: New Zealand and Australia	ICBT	Control: internet-based health information package	N=562	Depression symptoms	88% of participants completed the endpoint assessment at 12 weeks; ICBT was associated with a greater improvement in depressive symptoms versus control, with improvement increasing over time (p=0.012)
Johansson et al., 2019 [12]	CVD; Country: Sweden	ICBT	Control: moderated ODF	N=144	Depression, health-related QoL, depression-related function, pain, and adherence to medication	After seven modules of ICBT, depressive symptoms were significantly reduced compared with control conditions (p<0.001), utilising different scales, while mental ability, pain, and QoL were also improved (p<0.05); 60% of patients completed all modules.
Newby et al., 2017 [13]	Diabetes (type 1 or type 2); Country: Australia	ICBT, plus therapists available via email or phone support	TAU	N=91	Self-reported depressive symptoms, diabetes-related distress, glycaemic control, distress, disability, anxiety	At three months, ICBT was completed by 66% of participants and demonstrated superior improvements in depressive symptoms, anxiety, diabetes-related distress, and general distress; 87% of the iCBT group were no longer depressed at the end of the study, with 51% showing a clinical change compared to 18% in the TAU group.
Norlund et al., 2018 [23]	CVD (recent MI); Country: Sweden	Therapist-guided ICBT	TAU	N=239	Depression	All participants showed a reduction in depressive symptoms over 14 weeks, without differences between groups; adherence was low, and only 46.2% in the ICBT group completed the first module.
Wallert et al., 2018 [24]	MI	ICBT	Randomly control group	N=239	Self-assessed cardiac-related fear, anxiety, and sex	For developing and testing effective ICBT interventions, investigating factors that predict adherence is important. Adherence to iCBT for MI was predicted by cardiac-related fear and sex, consistency and verbal ability, or therapeutic alliance.
Lundgren et al., 2016 [25]	Heart failure; Country: Sweden	ICBT	Moderated ODF	N=50	Depressive symptoms, cardiac anxiety, QoL	After nine weeks of ICBT, depression symptoms significantly reduced; no differences were found between groups for other outcomes; the study was underpowered.
Van Bastelaar et al., 2011 [26]	Diabetes (type 1 or type 2); Country: Netherlands	ICBT	TAU	N=255	Depressive symptoms, diabetes-specific distress, glycaemic control	Depressive symptoms improved with ICBT (p=0.04), with 41% clinical improvement versus 24% in the control group (p<0.001)
Willems et al., 2016 [27]	Cancer survivors; Country: Netherlands	CBT-based Internet-tailored intervention	Waiting list control	N=462	Anxiety, depression, fatigue, QoL	At six months, depression was reduced with the intervention compared to the control group (p=0.007); positive findings were also noted for the intervention in terms of fatigue and emotional or social function compared to the control group.
Urech et al., 2018 [28]	Newly diagnosed with cancer; Country: Switzerland Austria, UK, Germany	CBT-based, Internet-tailored intervention	Waiting list control	N=222	QoL, distress, anxiety, depression	No differences were seen between groups for depression or anxiety outcomes after 12 weeks (p=0.15)
Dirkse et al., 2020 [29]	Cancer survivors; Country: Canada	ICBT	Self-guided or technician-guided CBT	N=86	Anxiety, depression, fear of cancer recurrence, QoL	All outcomes were consistently improved compared to the baseline, regardless of the delivery method of the ICBT.
Atema et al., 2019 [30]	Breast Cancer Survivor Country: Netherlands	ICBT	Waiting list control group	N=254	Sleep deprivation, QoL, hot flushes and night sweats	In comparison with the control group, ICBT with or without a therapist (p <0.001) has positive effects on hot flushes, sleep quality, and quality of life.
David et al., 2012 [31]	Haematologic cancer patients; Country: Germany	Online support programme based on CBT	Waiting list control	N=186	Psychological distress; mental adjustment	Assessment at baseline and after the four-week intervention showed an increase in "fighting spirit" compared to the waiting list control; no other differences were observed.
Boele et al., 2018 [32]	Cancer (glioma); Country: Netherlands	Online problem-solving therapy (based on CBT)	Waiting list control	N=89	Depressive symptoms, fatigue, health-related QoL	No difference was found between groups for depression scores after post-intervention, the three- or 12-month assessment.
Buhrman et al., 2015 [33]	Chronic pain; Country: Sweden	ICBT	Control: moderated ODF	N=52	Pain disability, depressive symptoms, anxiety symptoms	Pain, depression, and anxiety symptoms all improved to a greater extent in the iCBT group (p<0.05) after eight weeks of therapy; results were maintained at the one-year follow-up.
Day et al., 2019 [34]	Chronic low back pain; Country: Queensland, Australia	CBT-based cognitive therapy and mindfulness-based cognitive therapy (MBCT)	Mindfulness meditation (group)	N=69	Pain interference, depressive symptoms, physical function;, pain medication use	At three and six months, pain, depressive symptoms, and physical function improved across all groups (p<0.001); the only difference between groups regarding depression outcomes was suggested at six months (n=43). MBCT showed superior benefits for symptom reduction compared to mindfulness meditation.
Dear et al., 2013 [35]	Chronic pain	ICBT-based programme (clinician-guided)	Waiting list control	N=63	Depression, anxiety, pain, disability	All outcomes were improved with ICBT (five sessions over eight weeks) versus control, with effects persisting at the three-month follow-up.
de Boer et al., 2014 [36]	Nonspecific chronic pain; Country- Netherlands	ICBT	Waiting list control group	N=72	Pain-related catastrophizing, the intensity of pain, fatigue, pain-related interference, control, coping, global QoL and medical expenses	Better improvement was found in pain intensity in both ICBT and the face-to-face group sessions; and catastrophizing, pain coping, control and global QoL were better in internet sessions (two months booster). Moreover, iCBT was cost-effective.
Trompetter et al., 2015 [37]	Chronic pain; Country- Netherlands	ACT	Internet-based expressive writing (n=79) or waiting list control (n=77)	N=238	Pain, depression, psychological inflexibility	At three months, 48% improved significantly compared to the control groups regarding pain, depression, and psychological outcomes; 28% showed clinically significant improvement versus 5% in the control groups.

Methodological Quality

An overview of the critical findings of the CASP quality appraisal process is presented in Table 2. Overall, the studies showed a moderate level of methodological quality based on adherence to the principles of the RCT design. However, blinding was often a challenge, and attrition of patients during the study may also represent a significant risk of bias.

**Table 2:** Summary of the study quality Q: question The Critical Appraisal Skills Programme (CASP) 1. Did the study address a clearly focused research question? 2. Was the assignment of participants to interventions randomised? 3. Were all participants who entered the study accounted for at its conclusion? 4. Was the intervention blinded for participants, investigators, and analysts? 5. Were the study groups similar at the start of the trial? 6. Were the study groups treated equally? 7. Were the effects of the intervention reported comprehensively? 8. Was the precision of the estimate of the intervention or treatment effect reported? 9. Do the benefits of the experimental intervention outweigh the harms and costs? 10. Can the results be applied to your local population/in your context? 11. Would the experimental intervention provide greater value to the people in your care than any of the existing interventions? Responses: Yes (Y), No (N), I cannot tell (?)

Study	Q1	Q2	Q3	Q4	Q5	Q6	Q7	Q8	Q9	Q10	Q11
Glozier et al., 2013 [4]	Y	Y	Y	Y	Y	Y	Y	Y	?	Y	?
Johansson et al., 2019 [12]	Y	Y	Y	N	Y	Y	Y	Y	Y	Y	?
Newby et al., 2017 [13]	Y	Y	Y	N	Y	Y	Y	Y	?	Y	?
Norlund et al., 2018 [23]	Y	Y	Y	N	Y	Y	Y	Y	?	?	?
Wallert et al., 2018 [24]	Y	Y	Y	N	Y	Y	Y	Y	?	?	?
Lundgren et al., 2016 [25]	Y	Y	Y	N	Y	Y	Y	N	?	?	?
Van Bastelaar et al., 2011 [26]	Y	Y	Y	N	Y	Y	Y	Y	Y	?	?
Willems et al., 2016 [27]	Y	Y	N	N	Y	Y	Y	Y	?	Y	?
Urech et al., 2018 [28]	Y	Y	Y	N	Y	Y	Y	Y	?	Y	?
Dirkse et al., 2020 [29]	Y	Y	Y	N	Y	Y	Y	N	?	Y	?
Atema et al., 2019 [30]	Y	Y	Y	N	Y	Y	Y	Y	?	?	?
David et al., 2012 [31]	Y	Y	N	N	Y	Y	Y	N	?	?	?
Boele et al., 2018 [32]	Y	Y	Y	N	Y	Y	Y	Y	Y	?	?
Buhrman et al., 2015 [33]	Y	Y	Y	N	Y	Y	Y	Y	Y	?	?
Day et al., 2019 [34]	Y	Y	N	N	Y	Y	Y	Y	?	?	?
Dear et al., 2013 [35]	Y	Y	Y	N	Y	Y	Y	Y	?	Y	?
de Boer et al., 2014 [36]	Y	Y	Y	N	Y	Y	Y	Y	?	?	?
Trompetter et al., 2015 [37]	Y	Y	Y	N	Y	Y	Y	?	?	Y	?

Narrative synthesis

Cardiovascular Disease

The five studies suggested that an ICBT programme was beneficial in reducing depression symptoms using multiple measurement scales and was superior to online discussion forum participation. This effect was significant: 60% of patients in the ICBT arm of the study completed all seven modules, while 82% completed at least four [12]. A higher adherence rate was found in a study with 88% of patients completing all modules; the results suggested that ICBT was more effective than an online health information package in improving depressive symptoms over 12 weeks [4-8]. However, in some studies, researchers found that adherence was inadequate for the ICBT programme (only 46.2% of participants completed the first module), which may have accounted for the lack of difference between the ICBT and control groups regarding depressive symptom improvement [23]. In Wallert et al., the intervention comprised 14 weeks of therapist-guided and self-tailored ICBT, and some patients did not respond and were supervised by a licenced psychologist [24]. Increased adherence to ICBT was observed among self-assessed cardiac-related fear, sexual issues, and extended responses in their first homework assignment [24]. Finally, some studies fail to note any difference between the ICBT and control groups for depressive symptom improvement, potentially reflecting the underpowered nature of the study [25].

Diabetes

Two studies evaluated the use of ICBT in patients with diabetes, demonstrating the ICBT method's superiority. ICBT (six sessions over ten weeks) significantly reduced depressive symptoms, with 87% achieving remission at the three-month evaluation [13]. Similarly, other studies also show that clinical improvement was higher with ICBT than with usual care, suggesting consistency in the findings of these studies [23-26].

Cancer

Generally, studies showed that ICBT effectively promoted improvement in depressive symptoms in cancer survivors and patients with a cancer diagnosis undergoing therapy [27,28]. Cancer survivors showed improvement in depression and anxiety symptoms, quality of life, and fear related to cancer recurrence, whether ICBT was delivered in a self-guided or technician-guided format [29]. Atema et al. found that there was an improvement in comparison with the control group; the guided and self-managed ICBT groups reported a considerable decline in the perceived impact of hot flushes and night sweats (p <0.001 for both) as a long-term effect and improvements in sleep quality and depression [30]. ''Fighting spirit", a measure of psychological resilience, improved following an online CBT support programme in patients with hematologic cancer compared with a waiting list control. Other psychological factors linked to depression were also enhanced equally in both groups. The participants in both groups showed a high level of distress and symptom burden at baseline, potentially representing a difficult-to-treat group [31]. Boele et al. failed to note any impact of ICBT on depressive symptoms in patients with glioma, although this was a short-term study with a small sample size [32].

Chronic Pain

Outcomes of ICBT in the management of depression associated with chronic pain were consistent in suggesting that this form of therapy was superior to control when assessed over two to six months [33-35]. Convincing improvements were observed in areas including catastrophizing, pain tolerance, locus of control, and global health-related quality of life in the internet-based CBT group [36]. However, there was some variability in ICBT between patient groups; some ICBT was even more effective than others. All trials used ICBT in a standard format, except for some researchers who evaluated the use of acceptance and commitment therapy (ACT), which is a form of CBT, noting that this was superior in promoting clinically significant improvement in depression and pain symptoms compared to the waiting list or Internet-based expressive wiring control groups after three months [37].

Discussion

The findings of this review suggested that ICBT can improve depressive symptoms in patients with chronic diseases. The nature of ICBT varied across studies in terms of modules or treatment length and the design of the strategy, mainly as some online interventions were based on CBT, including acceptance and commitment therapy (ACT) and supportive approaches [38]. This heterogeneity did not have a noticeable effect on treatment outcomes. Therefore, the principles of CBT may be broadly applied and refined according to specific patient groups, highlighting the flexibility in the clinical application of this approach [39]. Self-guided or technician-guided CBT using online modules was another distinction among the interventions analysed. However, there was a lack of evidence strongly supporting one intervention over another, but Dirkse et al. found that the efficacy of face-to-face CBT (self-guided or technician-guided) was comparable to ICBT, suggesting that a range of delivery models may be equally effective with less travel time and avoid the stigma of proceeding to a therapist [29]. Further studies would be needed to confirm this and validate the optimal CBT delivery method in patient subgroups [40]. Furthermore, future research assessing optimal module content, the number of modules, and the need for support should also be considered to guide future practice [14]. However, the value of ICBT in targeting depressive symptoms was seen in most studies and suggested superiority to treatment as usual and other interventions in patients with chronic diseases, highlighting the clinical value of the approach.

Since chronic disease needs sustainable management, digital health interventions significantly enhance healthcare quality; however, most low-income countries still need to develop these approaches [41]. Digital interventions are accessible through mobile, computer, and tablet devices and improve social connections among people; access varies significantly worldwide, and the form of the interventions varies in different health settings [9]. The cognitive behavioural therapy elements most commonly seen in my included studies were behavioural activation (BA), activity scheduling, graded task assignment (GTA), problem-solving, relaxation therapy, cognitive restructuring, and relapse prevention. Nevertheless, other components are used, and the form of ICBT delivered can vary greatly. Our review found some improvement in primary outcomes but did not improve the secondary outcomes, including self-efficacy, acceptance, and catastrophizing, which are essential and may be critical in the long-term management of chronic health conditions [10].

Limitations of this review

One of the potential challenges in evaluating the efficacy of ICBT is that adherence to the modules or programme content can vary significantly. Although we evaluated good-quality RCTs, blinding was often a challenge, and attrition of patients during the study may also represent a significant risk of outcome bias. Furthermore, poor adherence may be challenging in online mental health programmes, including ICBT, and requires further research to explore possible barriers to online module completion and to support the use of motivational strategies, prompts, or reminders among frontline healthcare staff to encourage the uptake of these therapies [15]. There was significant heterogeneity between the studies included in this review. For example, patients at different stages of their chronic disease journey (i.e., immediately following an acute event or years following diagnosis) were included, and it is unclear if these groups may be comparable in terms of risk factors for depression and treatment response rates [10]. Albeit we aimed to evaluate depression associated with chronic disease, data were scarce regarding chronic kidney disease, COPD, and chronic liver disease, which might have impacted selection bias. Moreover, ICBT is unavailable in low- and middle-income countries, which limits our study findings and makes them not applicable across the world. Also, the infrastructure of ICBT varies; for example, there is a lack of trained mental health care professionals in lower-income countries [42]. Nonetheless, a further limitation in this review is the lack of peer-reviewed literature that compared different forms of ICBT or online therapies in a one-on-one setting, which limited the application to practise for physicians.

The impact of COVID-19

Mobile technology has seen a surge in usage with the COVID-19 pandemic, likely leading to sustained changes in healthcare delivery with a greater reliance on digital, mobile interventions. The adverse effects of COVID-19 are that it causes more harm to people with preexisting communicable and chronic diseases and affects many body systems [43]. Consultations by telemedicine, or "virtual clinics," help protect people from the pandemic [11]. More recent attention has focused on addressing mental health problems (depression, anxiety) faced by ordinary people due to the COVID-19 pandemic, which is made worse by isolation, loneliness, and social distancing [38]. Digital interventions show considerable promise because there is potential to provide continuous social and medical support by healthcare professionals, social workers, and volunteers [43]. Again, high-income countries like the USA, Sweden, and the United Kingdom, with better digital infrastructure, have an advantage with ICBT [38,43].

## Conclusions

This review suggests that ICBT effectively reduces depressive symptoms in patients with comorbid depression and chronic diseases, including CVD, cancer, diabetes, and chronic pain. The consistency of the effect of ICBT was noted across studies, although adherence to the intervention may influence its clinical efficacy. However, evidence showed that web-based CBT could help some groups of the population who are already privileged, and little evidence exists showing that ICBT can be used in lower-income countries, which can potentially increase global health inequities. Future research should address these shortcomings; long-term studies with diverse populations with chronic disease and depression are needed to explore facilitators for improving adherence to ICBT and optimising treatment effects.
